# Validation of an algorithm for automated classification of migraine and tension-type headache attacks in an electronic headache diary

**DOI:** 10.1186/s10194-020-01139-w

**Published:** 2020-06-12

**Authors:** Aaron Roesch, Markus A Dahlem, Lars Neeb, Tobias Kurth

**Affiliations:** 1 Institute of Public Health, Charité – Universitätsmedizin Berlin, Charitéplatz 1, 10117 Berlin, Germany; 2 Newsenselab GmbH, Blücherstraße 22, 10961 Berlin, Germany; 3 Department of Neurology with Experimental Neurology, Charité – Universitätsmedizin Berlin, Hindenburgdamm 30, 12203 Berlin, Germany

**Keywords:** Headache, Migraine, Tension-type headache, Classification, App application, Digital health, Algorithm, M-health, E-diary, M-sense

## Abstract

**Background:**

This study evaluates the accuracy of an automated classification tool of single attacks of the two major primary headache disorders migraine and tension-type headache used in an electronic headache diary.

**Methods:**

One hundred two randomly selected reported headache attacks from an electronic headache-diary of patients using the medical app M-sense were classified by both a neurologist with specialisation in headache medicine and an algorithm, constructed based on the ICHD-3 criteria for migraine and tension-type headache. The level of agreement between the headache specialist and the algorithm was compared by using a kappa statistic. Cases of disagreement were analysed in a disagreement validity assessment.

**Result:**

The neurologist and the algorithm classified migraines with aura (MA), migraines without aura (MO), tension-type headaches (TTH) and non-migraine or non-TTH events. Of the 102 headache reports, 86 cases were fully agreed on, and 16 cases not, making the level of agreement unweighted kappa 0.74 and representing a substantial level of agreement. Most cases of disagreement (12 out of 16) were due to inadvertent mistakes of the neurologist identified in the disagreement validity assessment. The second most common reason (3 out of 16) was insufficient information for classification by the neurologist.

**Conclusions:**

The substantial level of agreement indicates that the classification tool is a valuable instrument for automated evaluation of electronic headache diaries, which can thereby support the diagnostic and therapeutic clinical processes. Based on this study’s results, additional diagnostic functionalities of primary headache management apps can be implemented. Finally, future research can use this classification algorithm for large scale database analysis for epidemiological studies.

## Background

Despite being one of the most prevalent disorders worldwide, the two major primary headache disorders of migraines and tension-type headaches (TTH) remain underdiagnosed and undertreated in Europe and other parts of the world [1, 2]. The importance of distinguishing between migraine and TTH is threefold. First, guidelines recommend different drug therapies, second, changes in frequency and phenomenology over time can indicate chronic headache conditions, and medication overuse. Third, the automated classification of large sets of migraine and tension-type headache can shed some light on the co-occurrence of both conditions and the transformation of headache features during the progression into chronification or remission from chronic form. To increase specificity and sensitivity in the diagnosis of headache disorders, the International Classification of Headache Disorders (ICHD) has advanced to its current 3rd edition [3]. However, the correct application of this extensive set of evidence-based classification criteria can be time-consuming and challenging, especially in primary care settings [4].

Regarding treatments, patients with high severity of migraine and headache-related disability should receive acute and, if necessary, preventive migraine-specific therapy [5]. Yet, despite the international classification system, a significant barrier to optimal treatment remains the lack of accurate diagnosis, particularly for some forms of migraine. Take, for example, the difference between patients with episodic migraine and chronic migraines. Patients with episodic migraine often receive (86.7%) an accurate medical diagnosis [6]. The differential diagnosis used for episodic migraine involves a set of standard questions that elicits the general headache experience, combined with the traditional history taking, age of disease onset, treatments tried, and family history. In contrast, for chronic migraines only 24.6% of eligible patients received the correct chronic migraine diagnosis [7]. To diagnose the latter, headache days need to be counted for 3 months and each attack type needs to be classified. This more time-consuming querying of dozens of attacks over time and their systematic classification appears a barrier to correct diagnosis.

To improve diagnostic accuracy, doctors recommend that headache patients document in a diary their attacks and medication for evaluation in consultations [3, 8]. With recent shifts towards digital health technologies, computerized headache classification tools have been developed to help patients accurately document their attacks and to support physicians in making more informed decisions [9]. Generally, mHealth apps for headache disorders have increased in number and functionality over the past years [10]. However, the majority of commercially available mHealth apps in app stores lack validation and certification [11, 12]. Moreover, many pose severe privacy risks [13]. In Europe, new medical device regulation specifies that most medical apps will fall under a higher subclassification which require stricter software regulation [14]. Further, recent laws such as the digital care law in Germany will allow doctors to prescribe certified apps and reimbursement by public insurance [15]. Given this context, the need to accurately validate algorithms for use in mHealth apps is paramount.

To the best of our knowledge, no research has sufficiently investigated how to classify single headache events in electronic headache diaries. To resolve this need, in this paper, we present an algorithm that applies the ICHD-3 criteria to single headache events recorded in a migraine management app’s database. Our goal is to provide an efficient means to classify patient headache events as migraine or tension-type headache. Specifically, the aim being to investigate how accurately an algorithm classifies patient headache events as migraine or tension-type headache in electronic health diaries using ICHD criteria. As validation, both a neurologist specialised in headache medicine and the algorithm classified the headache-diary data from a medical apps’ database. Implementation of the tool tested in this study was in M-sense, an mHealth app for headache patients. Patients use this medical app for documenting headaches as well as potential trigger factors, all of which get summarized in reports for doctors. After 3 months of continuous data collection, the app visualizes correlations between headaches and tracked trigger factors, and it offers personalised behavioural therapy support.

What this study contributes is new knowledge to the field of personalised headache medicine, specifically regarding single headache event classifications using algorithms in electronic headache diaries. One clinical use case would be to facilitate the diagnosis of chronic migraine. Further, this study highlights how ICHD criteria can be operationalised and how electronic headache data can be leveraged.

## Methods

### Design of the algorithm

**Fig. 1:**
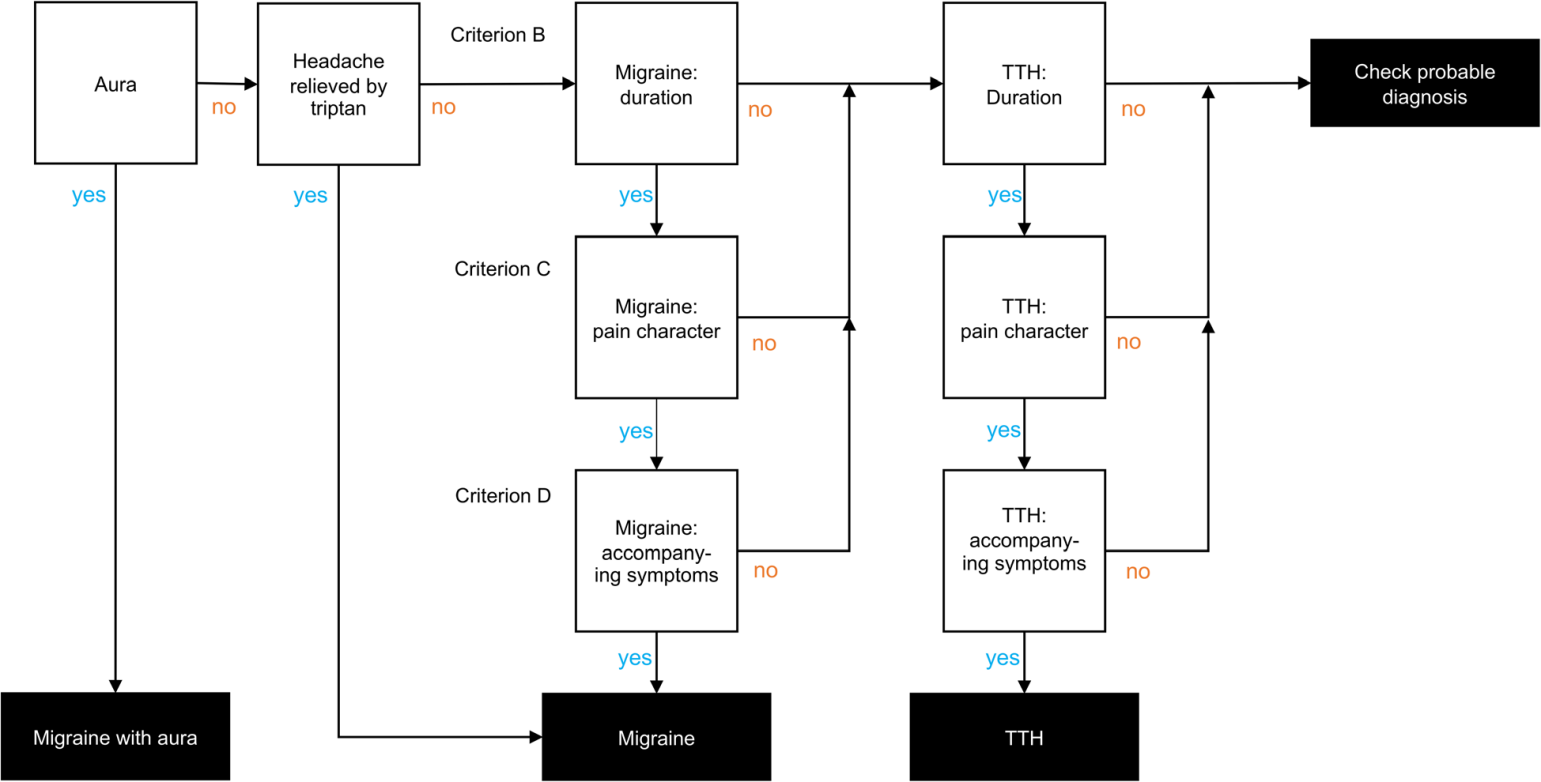
Schematic structure of the algorithm

**Fig. 2:**
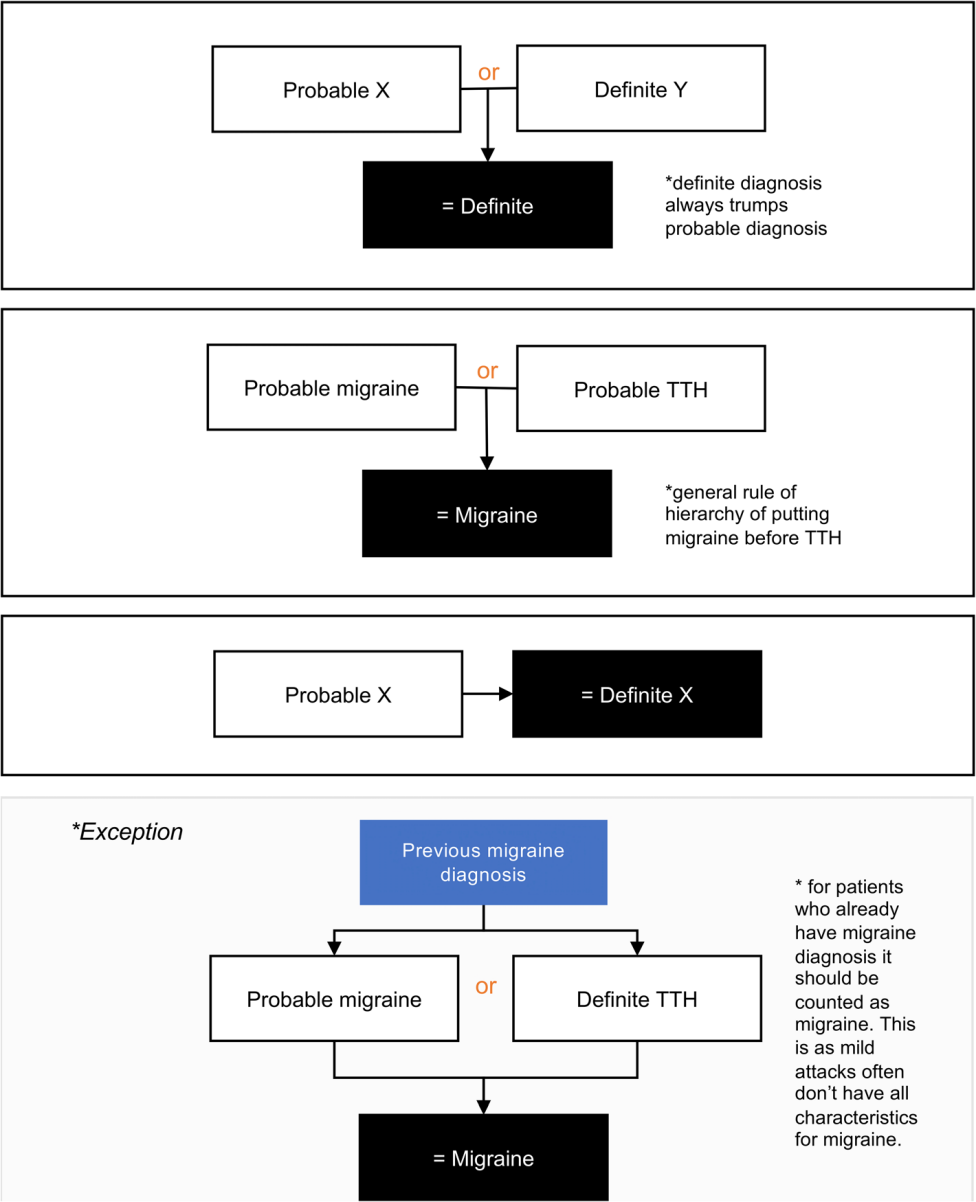
Algorithm rules for probable diagnosis

We developed an algorithm to classify primary headache disorders according to ICHD-3 criteria for both definite and probable Migraine without Aura, Migraine with Aura, and TTH as for usage in the M-sense app. The schematic structure of the algorithm is shown in Fig. 1 and algorithm rules for probable diagnosis is shown in Fig. 2. Although the ICHD-3 defines 30 different subtypes and subforms of migraines and 14 subtypes and subforms of tension-type headaches, it is not possible to map all of them in the algorithm. Moreover, “Trigeminal autonomic cephalalgias (TACs)” and “Other primary headache disorders” are not accounted for in the algorithm because they require more detailed anamnesis, a detailed neurological examination, or additional diagnostic tests. To identify if an attack should be counted as migraine or TTH we applied all relevant ICHD-3 rules. Classification differs depending on whether a patient has yet received a diagnosis. If yes, single attacks that fulfil criteria for probable migraine and TTH, should be counted as TTH, “under the general rule that definite diagnoses always trump probable diagnoses” [3]. Further the “general rule of hierarchy” puts migraine before TTH [3]. If to decide between probable migraine and TTH, for patients that have a previous migraine diagnosis, “probable migraine should be counted as migraine” because mild attacks may “not achieve all characteristics necessary for a migraine attack diagnosis but nevertheless respond to specific migraine treatments” [3]. Moreover, in the design of the algorithm, some adaptations to ICHD-3 criteria were necessary to translate the text-based ICHD-3 criteria into functional code. Adaption specifications are detailed below.

#### Pain intensity

Headache patients reported headache events in the app’s electronic diary, a structured procedure. Thereby data for pain intensity was assessed using the 11-point numeric rating scale (NRS-11). This contrasts to ICHD-3 which distinguishes pain on a 3-point verbal rating scale of mild, moderate, and severe. To accommodate for this scale difference, we translated the pain intensity into ICHD-3 criteria. The numeric rating scale assigned each number to its respective category: none (0), mild (1–3), moderate (4–6) and severe (7–10) [16].

#### Probable vs. definite diagnosis and other information

As previously stated, according to ICHD-3 criteria, definite diagnosis trumps a probable diagnosis. Exceptions are probable migraine, but only if there is a history of migraine for that patient. To decide between two possible probable diagnoses, other available information such as “the longitudinal headache history, the family history, the effect of drugs” should be considered [3]. Specifically, the algorithm also took into account cases where Triptans relieved symptoms, as this is part of diagnostic criteria C for chronic migraine. These cases were classified as migraine. Further, for the purpose of this study, headache attacks reported from patients using the app were classified as if to be from patients with a history of both migraine and tension-type headache. However, for privacy reasons this information and other relevant patient history was not stored in the app’s database.

#### Classifying single headache attacks

ICHD-3 criteria were originally designed to classify headache disorders as opposed to single headache attacks. Diagnostic criteria ‘A’ of the ICHD identifies that previously known attack occurrences and symptoms are important for diagnosis. Therefore, at least five attacks for migraine and ten attacks for TTH are required. In the algorithm design, criteria ‘A’ was accounted for through the headache history of patients, as mentioned previously. Criteria A was not further assessed for lack of necessity in single headache attack classification.

### Data collection

Data collection was between September 2016 and September 2017. From this data, a sample of 102 single headache events were randomly selected. For inclusion, users had to have consented to the app’s terms of service and data protection declaration, and headache attacks needed to have completed data entries. Excluded from this sample were thus incomplete data entries. The single headache event data collected was comprised of the start and endpoint of headache attacks, medication usage, as well as accompanying symptoms of pulsating pain, pressing pain, one-sided pain, pain on both sides, aggravation through physical activity, aura, vomiting, nausea, phonophobia, and photophobia. Patients entered this data using a combination of numeric scales and yes-no questions, either at the time of, or after a headache attack.

### Validation study

**Fig. 3:**
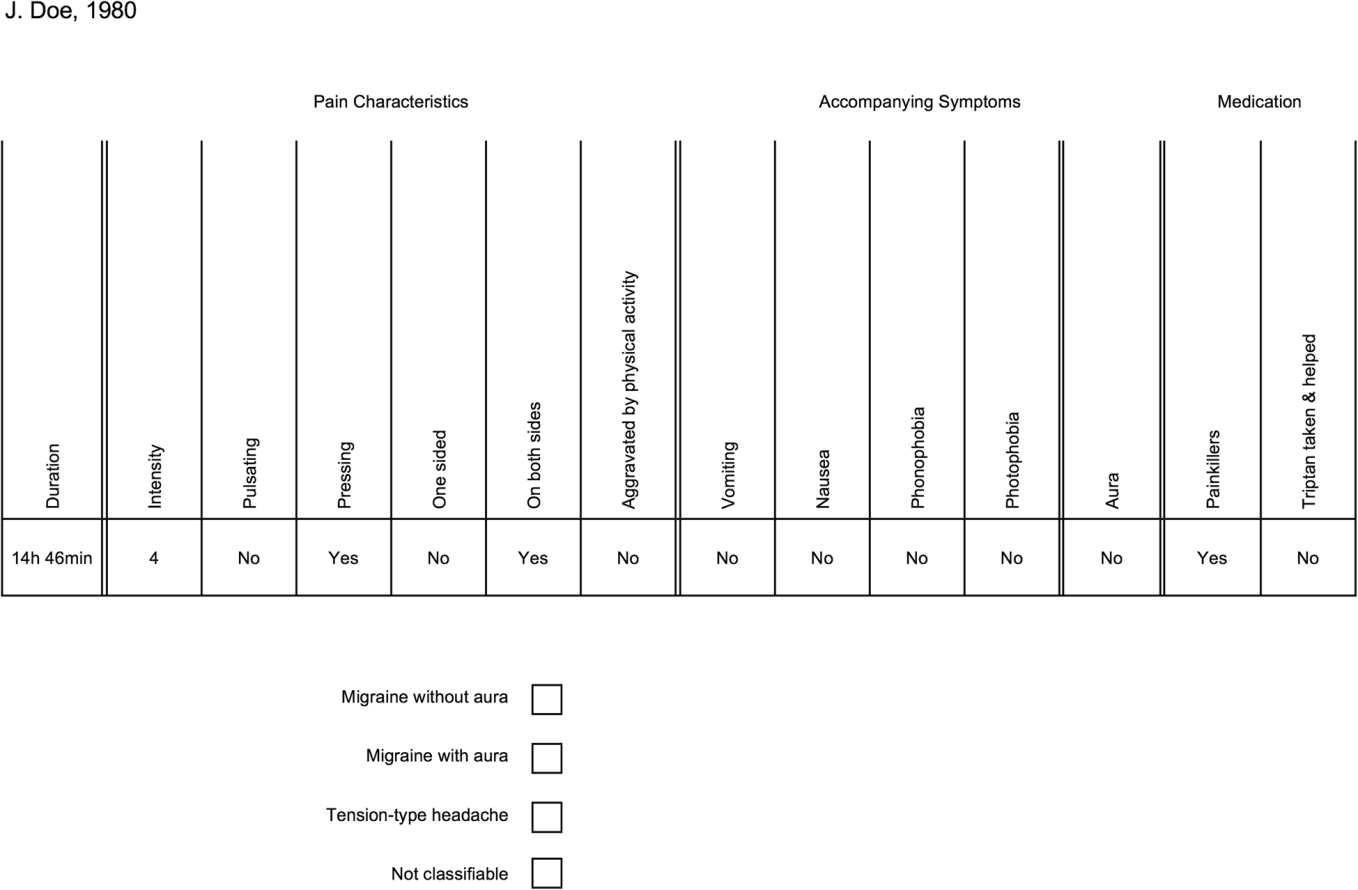
Generic single headache diary entry as seen by the neurologist

**Fig. 4:**
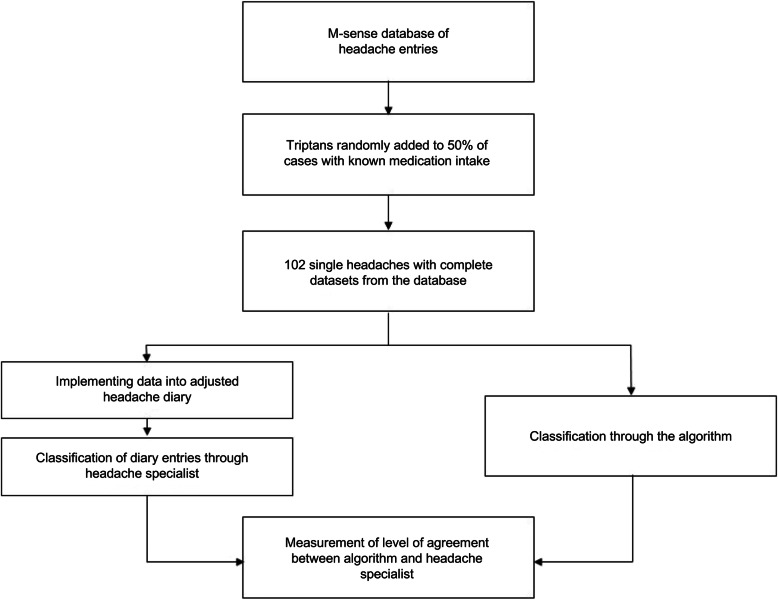
Flowchart for data collection and validation experiment

The validation study had two components: 1) an algorithm classification phase, and 2) a headache specialist evaluation phase. In the first phase, a computer-based algorithm based on ICHD-3 criteria was run and classified the 102 single headache events taken from the M-sense database. Results from this algorithmic classification were not available to the headache specialist. In the second phase of the validation study, the headache specialist classified the same 102 headache events also according to the criteria of ICHD-3 with information about an existing diagnosis of migraine and tension-type headache. To comply with clinical practice norms, the neurologist used a headache sheet for classification. This headache sheet, as shown in Fig. 3, was based on the headache calendar of the German Society for Headaches and altered to be consistent with the ICHD-3 classification criteria. Specifically, we included the variable ‘aggravation by physical activity’ while the criteria ‘odour sensitivity’ was excluded. Based on the evaluation using the headache sheet, the neurologist assigned the classification of migraine without aura (MO), migraine with aura (MA), TTH, or non-migraine or non-TTH (non-classifiable). We then compared results from the computer-based algorithm with the neurologist’s classification. The process is shown in Fig. 4.

### Data analysis

We calculated the kappa statistic to compare the algorithm’s classification results to each of the neurologist’s classifications based on the single-entry headache sheets. We evaluated the adequacy of the unweighted kappa coefficient (κ) using the following descriptive ranges: κ 0.40–0.59 is considered moderate, κ 0.6–0.79 substantial, and κ > 0.80 excellent agreement [17].

### Disagreement validity assessment

We conducted an in-depth disagreement validity assessment for cases of classification disagreement. First, we deduced disagreement categories from the recurring classification differences and symptom constellations. Based on these categories and the ICHD-3, a short questionnaire was developed, evaluated through discussion in the working group, and given to the neurologist to complete. Results from this questionnaire were then compared to the disagreement cases to identify why there was a difference in classification. Also, for all cases that the neurologist classified as non-identifiable further commentary was requested. We present the findings from this validity assessment in the below section of results.

## Results

### Validation study

Headache attacks of 102 patients were evaluated. Of these patients, 91 were female, and 11 were male, with an average age of 32 years and 31 years, respectively. The level of agreement between the neurologist and the algorithm’s classification of 102 single headache events resulted in 86 cases of agreement and 16 cases of disagreement. Meaning the unweighted kappa was κ = 0.74 [0.63, 0.86], representing a substantial level of agreement. Table 1 shows the details of the results. Table 2 shows features of the sample, which had an average headache duration of 13.5 h, and an average pain intensity of 4.8/10. The range of pain intensity was from 1 to 10 and the range of headache duration was 12 min - 4d 6 h 30 min.

**Table 1:** Cross-tabulation results and level of agreement between neurologist and algorithm

Cross Tabulation	MO (Algorithm)	MA (Algorithm)	TTH (Algorithm)	Not classifiable (Algorithm)	Total
MO (Neurologist)	48	0	1	1	50
MA (Neurologist)	0	14	0	0	14
TTH (Neurologist)	9	1	24	0	34
Not classifiable (Neurologist)	2	0	2	0	4
Total	59	15	27	1	102
Symmetric Measures:					
	Value	Asympt. Std. Error	~ T	~ Approx. Sig.	
Unweighted Cohen’s Kappa	.742	0.58	10.592	.00	
Number of valid cases	102

**Table 2:** Sample statistics

Sample feature	Frequency
average pain intensity	4.8/10
average headache duration	13.5 h
pain medication use	41%
pulsating pain	46%
pressing pain	54%
one-sided pain	56%
pain on both sides	44%
aggravation through physical activity	38%
aura	18%
vomiting	2%
nausea	25%
phonophobia	33%
photophobia	44%

### Disagreement validity assessment

Table 3 shows the six subcategories identified by the disagreement validity assessment. From the neurologist’s answers to the short questionnaire, we deduced that the algorithm correctly applied the ICHD-3 criteria in the 11 cases of category 1–4.

**Table 3:** Subcategories identified by the disagreement validity assessment

Origin of disagreement	Subcategory	Neurologist	Algorithm	n. of cases	Total
Inadvertent mistake	1	TTH	MO	7	12
	2	TTH (with Triptan)	MO	2	
	3	TTH	MA	1	
	4	MA	TTH	1	
	5	non-classifiable	MO or TTH	1	
Criterion E changes classification	5	non-classifiable	MO or TTH	3	3
Misinterpretation of ICHD-3	6	MA	non-classifiable	1	1

For subcategory five, in which the neurologist had categorized four cases as non-classifiable in contrast to the algorithm’s identification as MO or TTH, three of four of these cases had a short headache duration < 30 min in common. Commentary from the neurologist clarified that given the variety of possible diagnoses for short headache durations, classification was not possible without more detailed anamnesis. For the other case, the neurologist corrected his classification. For subcategory six, wherein the neurologist identified a case to be migraine without aura and the algorithm non-classifiable, we found that the algorithm was not coded to interpret the relevant ICHD criteria correctly. As such, in this case the algorithm could not detect probable diagnosis.

Consequently, we implemented a new rule to identify attacks with a short headache duration (< 30 min) and to mark them as “not classified” and to be evaluated separately. Furthermore, we adapted the code to apply ICHD criteria for probable diagnosis correctly.

## Discussion

Results from the current study demonstrate that the investigated algorithm for identifying headaches is a valid instrument for automated evaluation of electronic headache diaries. The study result of κ 0.74 [0.63, 0.86] indicates a substantial level of agreement and affirms that this algorithm, could, therefore support diagnostic and therapeutic clinical processes. In the case of classification disagreement, the algorithm more correctly applied the ICHD-3 criteria.

### Origin of disagreement

We identified three categories of disagreement in the disagreement validity assessment. In the first category, the neurologist inadvertently made mistakes. This error accounted for most of the disagreement cases (12 out of 16). This result is not surprising, given that the evaluation of a whole headache diary by classifying large numbers of individual attacks is a tedious task that requires high levels of concentration and does not reflect common clinical practice in headache diagnosis.

In the second category of disagreement, the neurologist considered the given information to be insufficient for accurate classification (3 out of 16). Criteria E in the ICHD-3 exists for this reason. It states, “Not better accounted for by another ICHD-3 diagnosis”, meaning that a neurologist would need to investigate further. For all cases in this second category, the headache duration was very short (< 30 min), so that many other ICHD-3 classifications such as “Trigeminal autonomic cephalalgias (TACs)” and “Other primary headache disorders” are potential differential diagnoses. Consequently, we added a rule to the algorithm to mark all headache attacks below 30 min duration.

In the third category, we misinterpreted ICHD criteria for probable diagnosis (1 out of 16), so that the algorithm applied rules too strictly and hence did not classify correctly. Specifically, a probable diagnosis was not identified as such if none of the criteria pain severity, quality, localisation, and aggravation by physical activity of the C-criterion applied to migraine.

Finally, the proportion of classification based on probable diagnosis was 38% (39/102). This may have contributed to disagreement between algorithm and neurologist as probable diagnosis adds another layer of complexity to classification.

### Discussion of the algorithm

The kappa 0.74 is a good outcome, particularly considering uncertainties associated with the clinical diagnosis of migraines. For example, one study identified that agreement between neurologists asked to assign a headache diagnosis based on the review of videotaped patient interviews, ranged in a kappa from 0.55 to 0.81 [18].

The information from an entry in the headache diary (Fig. 3) represents a symptom complex and is a complete data set for distinguishing migraine from TTH. It is the task of the physician to transform this subjective evidence into an accurate diagnosis. Since migraine and TTH themselves are phenomenological diagnoses, other possible diagnoses, such as secondary headaches, must be excluded via differential diagnosis which is reflected by the criterion E in ICHD-3. Additionally, criterion A in ICHD-3 defines the number of attacks or headache days that are necessary before a diagnosis can be made [3]. Therefore, this study evaluates only the classification of single attacks, not the diagnosis of a patient.

As mentioned in the method section, classification also depends on previous classification and to correctly assign these attacks the diagnosis made by a doctor should be added to the app’s profile. Such specifications can be added when a doctor prescribes a certified app for their patients.

“Characterization of frequently recurring headache generally requires a headache diary” [3], to record headache related symptoms and to count the number of headache days for differential diagnosis of episodic and chronic headache. A headache patient usually presents a headache diary during a doctor’s consultation, which consists of individual diary entries, for example listing 31 rows for each day of a month [19]. The number of headache days is a criterion for the diagnosis of chronic headache and several attacks recorded on 1 day, are still counted as one headache day. Therefore, to be a more useful tool for clinicians, both the number of headache attacks and headache days can be displayed in the app.

### Comparison to other computerized headache classification or diagnostic systems

Several studies evaluate the use of computerized headache classification or diagnostic (CHD) systems. Andrew, Penzien [20] were one of the first to find that their CHD system provided a general improvement in headache classification reliability. More recently, De Simone, Coppola [21], validated the AIDA Cefalee diagnostic expert system as a reliable diagnostic tool for primary headaches. This system was based on ICHD 2 criteria and intended for use by physicians. Similarly, the CHD system was validated to account for all primary headaches [22]. Of note, is also Dong, Yin [23] validation study of a guideline-based CHD system. Their results reported the system had good accuracy. Further, is the Computerized Headache Assessment Tool (CHAT) designed and validated to identify several primary headache disorders, including episodic and chronic migraine [24]. Kaiser, Igdalova [25] evaluated the Penn Online Evaluation of Migraine (POEM) instrument, which follows a questionnaire branching logic and suggests its application for research. Different approaches to classify headaches also include optimization algorithms [26] or other machine learning algorithms, which we didn’t contemplate given the rule-based structure of ICHD-3.

Therefore, the results of this study build on existing evidence that algorithms can classify headaches. Further, the study is unique because it focuses on the classification of single headache events, which is the single unit headache diaries get based on. This approach thus allows meaningful integration into mHealth apps, intelligent headache diaries, and pre-interpreted patient reports as decision support tools for doctors that also improve patient understanding.

### Limitations

It is beyond the scope of this study to diagnose migraine or TTH as a diagnosis should only be made after a physician visit. Further, it is not possible to do so using the limited data available in headache diaries. Instead, this study focused on the classification of single headache attacks.

Commonly used in clinical practice as a diagnostic criterion for migraines is the efficacy of triptans in headache attacks. However, the ICHD-3 only considers the efficacy of a triptan in the identification of a migraine day in chronic migraine [3]. Nonetheless, the time criterion 4–72 h in the classification of migraine attacks is only valid for “untreated or unsuccessfully treated” [3] attacks. At the time of validating the algorithm, the database did not store the type of pain medication taken for each attack and therefore had no record of whether the respective medication was a triptan or not. To solve this issue, we added the feature ‘triptan taken and effective’ to 50% of all headaches with a known medication intake for the experiment, which is a limitation to the validity of the data.

Further limitations in the data collection include the assessment of an aura through a yes-no question. Also, that users of the app were able to read about auras in an informational text. The reliability of collected data was thereby impacted because it depended on users correctly recognizing and identifying auras according to diagnostic criteria, which includes certain symptoms and six additional characteristics. Tools like the “visual aura analogue scale” could be a solution for this issue [27]. However, for usability concerns, this tool was not implemented.

Regarding the generalisability of results, as the data-set used in this study was derived from the M-sense database, this data may not be representative of the wider population and may have a selection bias towards a more tech-savvy population. In regard to gender bias, as gender is not a criteria of ICHD-3, the marked gender difference in the data set does not impact the study results.

Another methodological limitation was that the neurologist did not have the possibility to ask further questions or access more detailed information about medical history of the patient, defined as the gold standard in migraine diagnosis. Possible study limitations, therefore, also include using a neurologist with limited access to information as a reference test.

### Future research

Further studies should investigate the application of automated electronic headache diaries. Headache diaries already have various benefits and may help expand the knowledge about headache disorders. They can: help differentiate between migraine and TTH [8]; reduce recall bias [28]; help diagnose more than one headache type in a patient; help differentiate between episodic and chronic headache; and identify triggers [29, 30]. Despite obvious benefits, compliance with paper diaries is low [31], but can be improved in electronic diaries with compliance enhancing features such as reminders [32]. Making electronic headache diaries more intelligent through the implementation of algorithms increases their functionalities and can help develop more personalized therapies such as pre-emptive therapy [33]. Further, they can inform or even drive clinical management in various ways [34]. Another area of application is the identification of digital biomarkers for companion diagnostic [35, 36].

Further research should also explore the potential of working with real-world data of large headache databases such as M-senses’. These databases could help to investigate the controversial question of whether tension-type headaches are their own disease entity or rather a severity continuum [37, 38]. Finally, further research could use cluster analysis on headache data to research the most suitable headache criteria and its potential for the next ICHD edition [39, 40].

## Conclusion

The results of this study confirm the accuracy of an algorithm for automated classification of MA, MO, and TTH, with a substantial level of agreement to a neurologist specialized in headache medicine. Hence, the tool is a suitable instrument for automated evaluation of electronic headache diaries, to facilitate the error-prone and time-consuming manual evaluation and can thereby facilitate the diagnosis of chronic migraine. Based on this study’s results, additional diagnostic functionalities of headache management apps can be implemented. However, this study demonstrates that an automated evaluation is only useful in conjunction with a doctors’ examination. Future research can use this classification algorithm for large scale database analysis for epidemiological studies, for example to investigate whether migraine and tension-type headache are diagnostic types or points on a severity continuum [37].

## Data Availability

The datasets used and analysed during the current study are available from the corresponding author on request.
